# Synthesis, antibacterial and antiproliferative potential of some new 1-pyridinecarbonyl-4-substituted thiosemicarbazide derivatives

**DOI:** 10.1007/s00044-016-1599-6

**Published:** 2016-06-01

**Authors:** Monika Pitucha, Maciej Woś, Malgorzata Miazga-Karska, Katarzyna Klimek, Barbara Mirosław, Anna Pachuta-Stec, Agata Gładysz, Grazyna Ginalska

**Affiliations:** Department of Organic Chemistry, Medical University, Chodzki 4A, 20-093 Lublin, Poland; Chair and Department of Biochemistry and Biotechnology, Medical University, Chodzki 1, 20-093 Lublin, Poland; Department of Crystallography, Faculty of Chemistry, Maria Curie-Sklodowska University, pl. M. Curie-Skłodowskiej 3, 20-031 Lublin, Poland; Department of Medicinal Chemistry, Medical University of Lublin, Jaczewskiego 4, 20-090 Lublin, Poland

**Keywords:** Thiosemicarbazide, Antibacterial activity, Cytotoxicity, Cell proliferation

## Abstract

In this study, the antibacterial, cytotoxic and antiproliferative activities of novel thiosemicarbazide derivatives were assessed. Our results demonstrated that some of the novel compounds possess good antibacterial properties against *Staphylococcus epidermidis*, *Streptococcus mutans* and *Streptococcussanguinis* and are only slightly cytotoxic; thus, they exhibit an excellent therapeutic index, which is higher than that of ethacridine lactate. Moreover, our data showed that compounds **2** and **4** have an antiproliferative activity against human breast adenocarcinoma and human hepatocellular carcinoma cell lines. We expect that the novel thiosemicarbazide derivatives can be used as agents for treatment of dental caries and also for chemotherapy support.

**Electronic supplementary material:**

The online version of this article (doi:10.1007/s00044-016-1599-6) contains supplementary material, which is available to authorized users.

## Introduction

For many years, new drugs of an interesting structure, unknown molecular target, low toxicity and a high therapeutic index have been looked for. This is due to the impossibility of treating many serious diseases, such as bacterial infections or cancer. For a few years, the attention of researchers has been focused on thiosemicarbazide derivatives, which were investigated as a pharmacophore for antimicrobial and anticancer activity (Salgın-Gökşen *et al.*, 2007). In vitro screening of some thiosemicarbazides demonstrated activities against *Escherichia coli*, *Klebsiella pneumoniae* (recultured), methicillin-resistant *Staphylococcus aureus*, methicillin-sensitive *Staphylococcus aureus* and *Mycobacterium tuberculosis* (Sheikly *et al.*, 2012; Umadevi *et al.*, 2012; Patel *et al.*, 2014; Tan *et al.*, 2012). Many of the compounds showed a good antibacterial activity against *K.pneumoniae* (Alagarsamy *et al.*, 2010) and *S. aureus* in comparison with the standard drug—ciprofloxacin (Rane *et al.*, 2014). Additionally, thiosemicarbazides are one of the most promising biologically active compounds which can be used in cancer treatment (Arora *et al.*, 2014; Mohsen *et al.*, 1981). These derivatives have been effectively used against a number of carcinoma cell lines (Perković *et al.*, 2012; Bhata *et al.*, 2008; Malki *et al.*, 2014; Zhang *et al.*, 2011). It has been found that thiosemicarbazide derivatives demonstrated cytotoxic and antiproliferative activity against HeLa, HepG2, MDA-MB-231 and HT-29 cell lines (Mavrova *et al.*, 2014).

In this study, we synthesized new thiosemicarbazide derivatives and investigated their antibacterial, cytotoxic and antiproliferative properties. We expected the presence of the pyridine ring to significantly affect the biological activity of the tested derivatives. Additionally, because the literature lacks information about the effectiveness of thiosemicarbazide derivatives against oral bacteria, we decided to perform in vitro tests against *Streptococcus mutans* and *Streptococcus sanguinis*. It is extremely important because bacterial infections co-occurring with dental caries may be the cause of chronic diseases such as endocarditis, myocardial infarction (Cognasse *et al.*, 2014; Kerrigan *et al.*, 2002) and cancer, for example, pancreatic and gastrointestinal cancer (Meurman, 2010). It is worth highlighting that cancer patients undergoing chemotherapy often suffer from oral complications.

## Results and discussion

### Chemistry

**Scheme 1:**
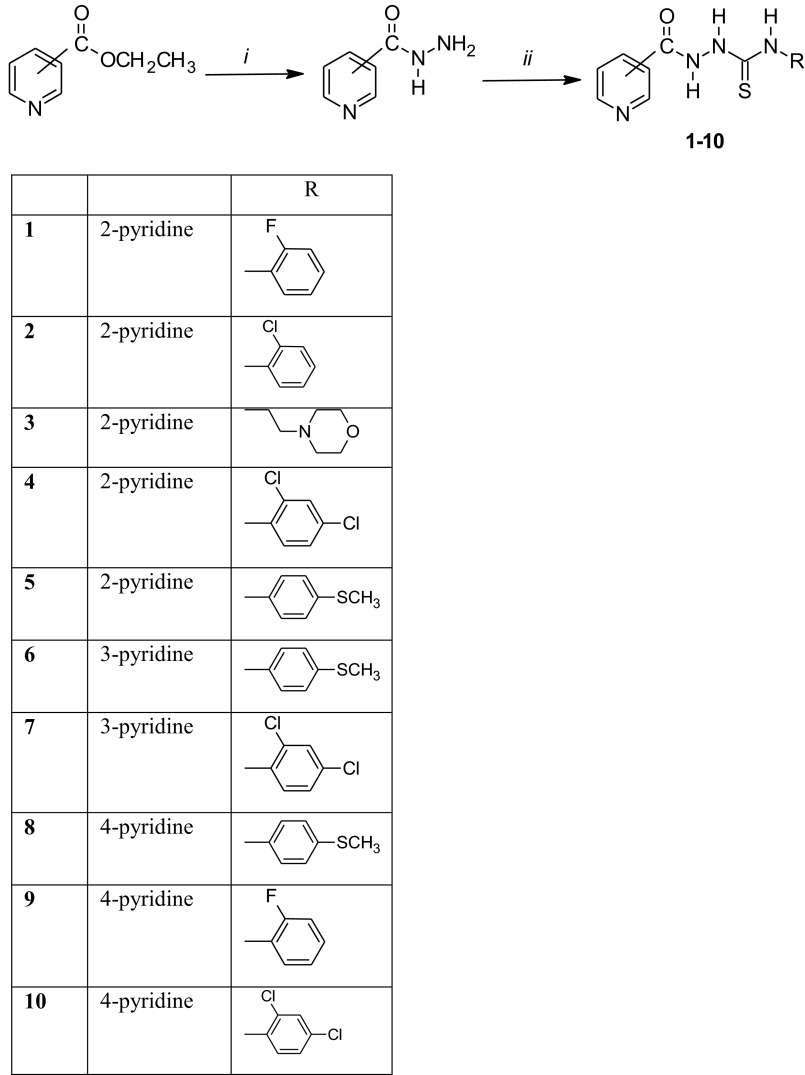
Synthesis 1-pyridinecarbonyl-4-substituted thiosemicarbazide derivatives. Reagents and conditions: (i) NH_2_NH_2_·H_2_O, C_2_H_5_OH, reflux; (ii) RNCS, CH_3_OH, reflux

The 1-pyridinecarbonyl-4-substituted thiosemicarbazide derivatives (**1–10**) were prepared by the reactions of 2-, 3- or 4-pyridine carboxylic acid hydrazide with isothiocyanates. The reaction was carried out in methanol as solvent and was refluxed for 30 min. The synthesis of all compounds was accomplished using the reaction illustrated in Scheme 1.

Compounds **9** and **10** were obtained earlier (Byung *et al.*, 2004; Goldfarb, 2009). According to the Chemical Abstracts Service (SciFinder), some of the compounds (**1–4** and **7**) have the CAS number, but there is no method synthesis and references.

The structures of obtained compounds were confirmed by spectral analysis (^1^H, ^13^C NMR, IR and MS). For compound **2** was performed X-ray diffraction analysis.

**Fig. 1:**
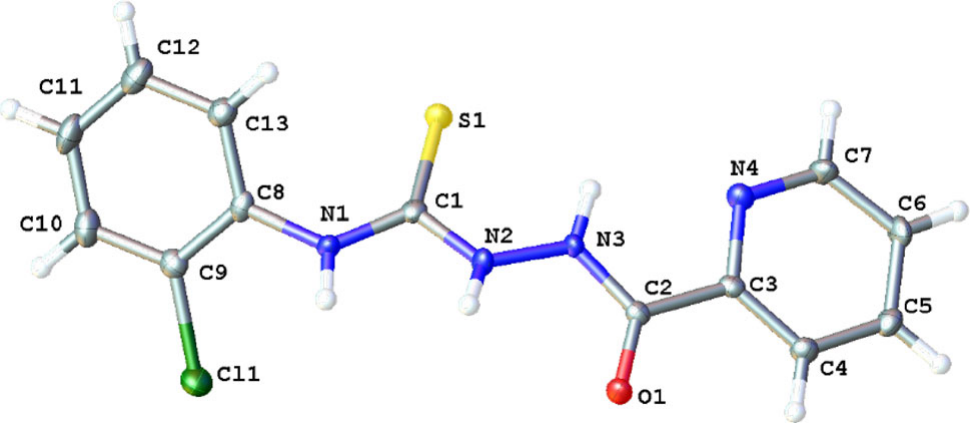
Molecular structure with atom numbering scheme for compound **2**

**Fig. 2:**
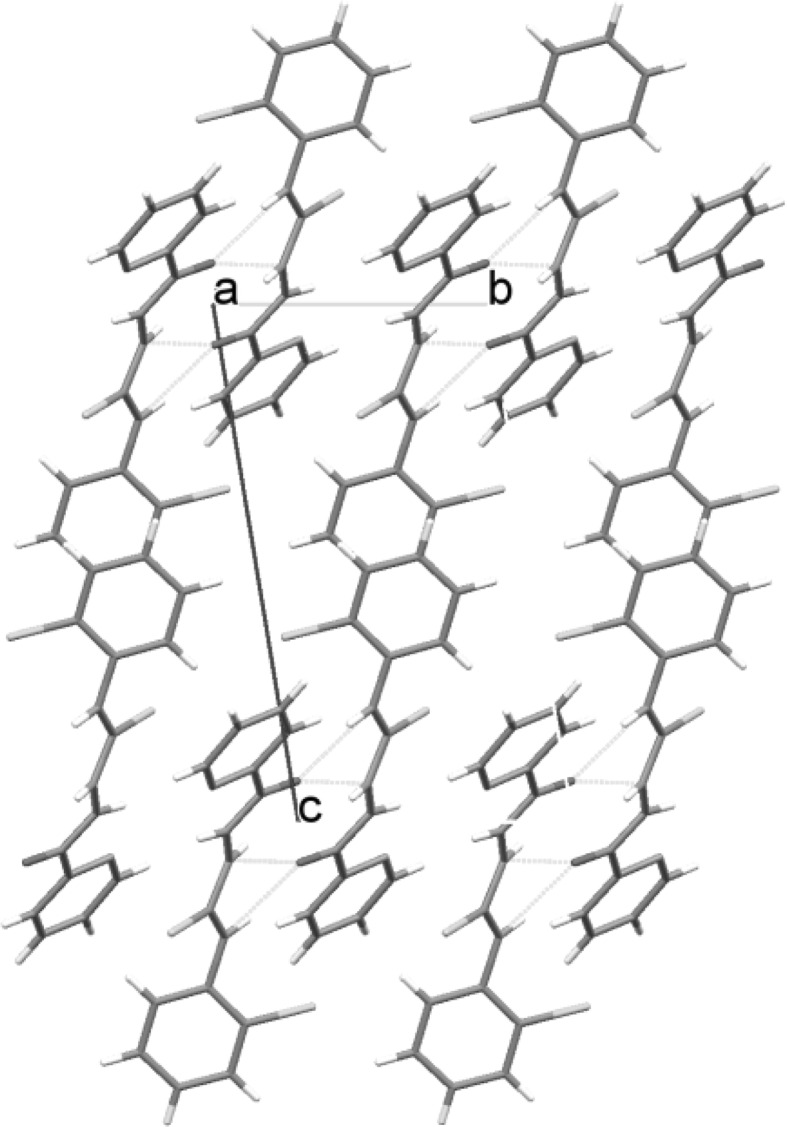
Crystal packing in compound **2**

Figure 1 shows that this compound crystallizes in the triclinic *P*-1 space group. The molecule of compound **2** has an extended but not planar conformation with the dihedral angle of 47° between the mean planes of aryl rings. In this compound, molecules interact through two N–H…O hydrogen bonds (*d*_N1…O1_ = 2.860(3) Å, ∠_N1–H1…O1_ = 153(4)° and d_N2…O1_ = 2.800(4) Å, ∠_N2–H2…O1_ = 141(4)°) forming dimers. Between the chlorophenyl rings, there is a *π*…*π* interaction with a distance between the ring centroids of 3.728(4) Å. The molecules stack in columns along the *a* axis (Fig. 2).

### Antibacterial activity evaluation

All synthesized compounds were initially screened for their potential in vitro antibacterial activity using the agar dilution technique. It was found that seven out of ten thiosemicarbazide derivatives (**1**, **2**, **4–7**, **10**) effectively inhibited some of the tested strains (Table 1).

**Table 1:** Zones of bacterial growth inhibition (mm) produced by 100 μg for the more active compounds and standards

Compound	Zone of bacterial growth inhibition (mm)							
	*S.a*	*S.e*	*E.c*	*P.a*	*S.m*	*S.s*	*L.a*	*L.*spp.
**1**	18	26	22	12	10	10	12	24
**2**	17	25	20	4	24	0	16	24
**3**	0	7	0	0	0	0	0	0
**4**	22	27	20	14	35	25	20	24
**5**	10	10	11	0	0	15	19	11
**6**	10	9	9	0	11	19	0	0
**7**	0	10	9	0	24	10	5	7
**8**	0	0	0	0	0	0	0	0
**9**	0	0	0	0	0	0	0	0
**10**	16	13	7	0	28	20	0	0
Cefepime	38	36	35	29	32	29	31	29
CLX	23	23	15	16	26	29	24	24
Ethacridine lactate	10	8	12	4	24	24	15	10

*S.a*—*Staphylococcus aureus* ATCC 25923, *S.e*—*Staphylococcus epidermidis* ATCC 12228, *E.c*—*Escherichia coli* ATCC 25922, *P.a*—*Pseudomonas aeruginosa* ATCC 9027, *S.m*—*Streptococcus mutans* PCM 2502*, S.s*—*Streptococcus sanguinis* PCM 2335, *L.a*—*Lactobacillus acidophilus* PCM 210*, L.*spp.—*Lactobacillus* spp.

Two thiosemicarbazides (**1**, **4**) showed potential activity against all tested aerobic Gram-positive, aerobic Gram-negative and microaerobic Gram-positive bacterial strains. Additionally, zones of bacterial growth inhibition of some compounds were higher compared with CLX and ethacridine lactate. Only cefepime was characterized by large zones of inhibition (29–38 mm) in comparison with thiosemicarbazide derivatives. The detailed in vitro antibacterial activity of the potentially active compounds was later determined using the broth microdilution method on the basis of minimal inhibitory concentration (MIC). Six of the compounds (especially **1**, **2**, **4**, but also **5**, **6**, **10**) had a potential activity against aerobic Gram-positive bacteria (MIC = 15.6–500 μg/mL). The antimicrobial activity of derivatives **1**, **2** and **4** against these bacteria was greater or similar to the activity of the control ethacridine lactate. The same compounds (**1**, **2**, **4**, **5** and **6**) were also found to effectively inhibit the growth of Gram-negative *E. coli* at a concentration between 62 and 125 μg/mL. The growth of *Pseudomonas aeruginosa* was moderately inhibited only by compounds **1** and **4** (MIC = 500 μg/mL for both) (Table 2).

**Table 2:** Minimum inhibitory concentration (MIC [μg/mL]) of the tested compounds against bacterial strains

Compound	*S. aureus*	*S. epidermidis*	*E. coli*	*P. aeruginosa*	*S. mutans*	*S. sanguinis*	*L. acidophilus*
**1**	31.25	15.6	62.5	500	250	500	125
**2**	31.25	31.25	62.5	1000	250	250	62.5
**3**	NA	NA	NA	NA	NA	NA	NA
**4**	31.25	31.25	62.5	500	31.25	7.81	31.25
**5**	250	125	125	>1000	31.25	31.25	500
**6**	250	500	62.5	>1000	250	15.63	>1000
**7**	>1000	500	>1000	>1000	500	1000	>1000
**8**	NA	NA	NA	NA	NA	NA	NA
**9**	NA	NA	NA	NA	NA	NA	NA
**10**	125	250	1000	1000	250	1000	1000
Cefepime	0.976	0.976	0.015	0.488	0.122	0.122	3.9
CLX	0.488	0.488	0.488	15.6	0.488	7.81	0.976
Ethacridine lactate	31.25	31.25	15.63	125	62.5	31.25	31.25

NA means inactive

The thiosemicarbazide derivatives (**1**, **2**, **4**, **5**) showed significant activity (MIC = 7.81–500 μg/mL) against the tested pathogenic microaerobic bacteria (causing dental caries). The strongest antibacterial properties were exhibited by compound **4**, whose MIC was 7.81 μg/mL against *S. sanguinis* and 31.25 μg/mL against *S. mutans*, *Lactobacillus acidophilus* and *Lactobacillus* spp. Substance **6** also showed significant activity against the pathogenic oral bacteria *S. sanguinis*, *S. mutans* but simultaneously did not limit the growth of the probiotic dental flora: *L. acidophilus*, *Lactobacillus* spp. These data suggest the possibility of using compounds especially **6** and **4** as well as **1** and **2** in the treatment of caries (Table 2). It is worth noting that among the tested pyridine derivatives, compounds **4** and **5** showed greater or equal activity against *S. mutans* and *S. sanguinis*, and compound **6** against *S. sanguinis*, than the commonly used antiseptic ethacridine lactate. Additionally, the activity of compound **4** against *S. sanguinis* was equal to that of chlorhexidine (CLX) (MIC = 7.81 μg/mL). CLX is an antiseptic drug used in the prophylaxis and treatment of dental caries (Autio-Gold 2008). However, the in vitro activity of the newly synthesized compounds against other tested bacterial strains was found to be lower compared to the controls (Cefepime, CLX and ethacridine lactate).

### Cytotoxic activity evaluation

**Fig. 3:**
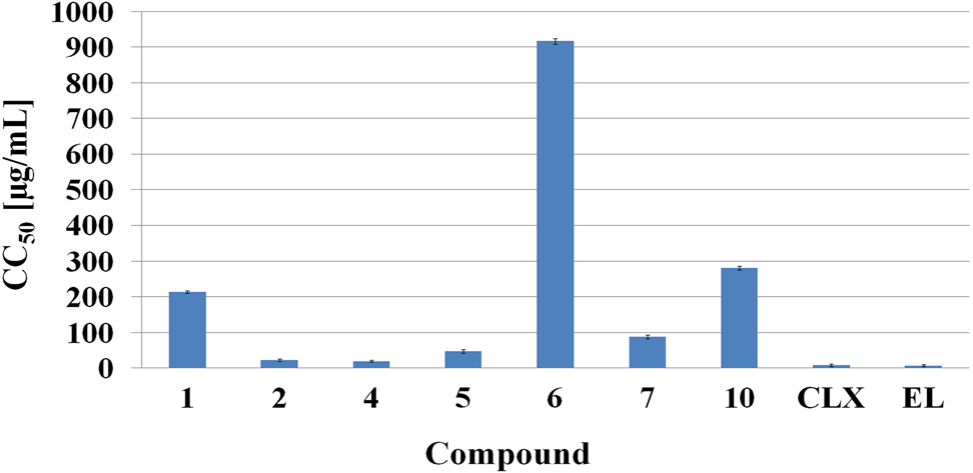
In vitro cytotoxic activity (CC_50_) of the compounds against normal human skin fibroblasts. The data were expressed as mean values ± SD from three independent experiments. *CLX* Chlorhexidine, *EL* Ethacridine lactate

The synthesized compounds and the reference antibacterial agents were evaluated for the in vitro cytotoxic activity against the BJ cell line (normal human skin fibroblasts) using the MTT assay. The calculated response parameter was CC_50_, which corresponds to the concentration required for a 50 % reduction of cell viability. The in vitro cytotoxic activity of the synthesized compounds and the reference antibacterial agents is summarized in Fig. 3.

In the cytotoxic study, the novel thiosemicarbazide derivatives showed significant differences in cytotoxicity. The CC_50_ values of the synthesized compounds ranged from 19.5 to 917.4 μg/mL. Among all tested compounds, 4-(2,4-dichlorophenyl)-1-(pyridin-2-yl)carbonylthiosemicarbazide (**4**) exhibited the highest cytotoxic activity with a CC_50_ value of 19.5 μg/mL. Nevertheless, compound **4** showed lower cytotoxicity than the two reference antibacterial agents (CLX and ethacridine lactate), whose CC_50_ values were 8.46 μg/mL and 6.88 μg/mL, respectively. It is worth noting that 4-(4-methylthiophenyl)-1-(pyridin-3-yl)carbonylthiosemicarbazide (**6**) only slightly decreased the BJ cell viability with a CC_50_ value of 917.4 μg/mL. This result indicated that compound **6** showed the lowest cytotoxic activity in comparison with both the tested thiosemicarbazide derivatives and the reference antibacterial agents. Most interestingly, 4-(2,4-dichlorophenyl)-1-(pyridin-3-yl)carbonylthiosemicarbazide (**7** with a CC_50_ of 88.3 μg/mL) significantly decreased cell viability compared to compound **6**. It was not possible to calculate the CC_50_ value for cefepime as it did not reduce cell viability by 50 % at the highest tested concentration of 1500 μg/mL (data not shown). It is widely known that all drugs applicable in the treatment of bacterial infections should exhibit high antibacterial efficiency and low toxicity toward human cells. Thus, many researchers claim that the profile of in vitro cytotoxicity of antibacterial agents may be characterized by the CC_50_/MIC ratio (Kashyap *et al.*, 2012; Panchal *et al.*, 2009; Zoraghi *et al.*, 2011). For this reason, in the present study, we attempted to evaluate the in vitro therapeutic potential of novel thiosemicarbazide derivatives and compared them to the reference antibacterial agents (Table 3).

**Table 3:** In vitro therapeutic potential of the compounds

Compound	TI							
	Bacterial species							
	*S.a*.	*S.e*.	*E.c.*	*S.m*.	*S.s.*	*L.a*.	*L.*spp.	*P.a*.
**1**	6.82	13.70	3.41	0.85	0.43	1.70	6.83	0.43
**2**	0.72	0.72	0.36	0.09	0.09	0.36	0.72	ND
**3**	ND	ND	ND	ND	ND	ND	ND	ND
**4**	0.62	0.62	0.31	0.62	2.50	0.62	0.62	0.43
**5**	0.19	0.38	0.38	1.53	1.53	0.10	0.09	ND
**6**	3.67	1.83	14.68	3.67	58.70	ND	ND	ND
**7**	ND	0.18	0.08	0.18	0.08	ND	ND	ND
**8**	ND	ND	ND	ND	ND	ND	ND	ND
**9**	ND	ND	ND	ND	ND	ND	ND	ND
**10**	2.25	1.12	ND	1.12	0.56	0.28	ND	ND
Chlorhexidine (CLX)	17.33	17.33	17.33	17.33	1.08	8.66	34.6	0.54
Ethacridine lactate	0.22	0.44	0.44	0.11	0.44	0.22	ND	ND

TI (therapeutic index): the ratio between CC_50_ and MIC values, ND means not determined, due to lack of CC_50_ or MIC values, *S.a*—*Staphylococcus aureus* ATCC 25923, *S.e*—*Staphylococcus epidermidis* ATCC 12228, *E.c*—*Escherichia coli* ATCC 25922, *P.a*—*Pseudomonas aeruginosa* ATCC 9027, *S.m*—*Streptococcus mutans* PCM 2502*, S.s*—*Streptococcus sanguinis* PCM 2335, *L.a*—*Lactobacillus acidophilus* PCM 210*, L.*spp.—*Lactobacillus* spp.

The TI values below 1 obtained by the tested substances correspond to the lack of therapeutic safety. Among the synthesized compounds, derivatives **1**, **6** and **10** showed the highest values of therapeutic index. Compound **6** exhibited the in vitro therapeutic potential against *S. aureus*, *S. epidermidis*, *E. coli* and, which is important, against *S. mutans* and *S. sanguinis*, with the TI values of 3.67, 1.83, 14.68, 3.67 and 58.7, respectively. The essential observation is that the in vitro therapeutic indices of compound **6** were approximately 4–133 times higher than the in vitro TI values of ethacridine lactate and 58 times higher than the TI value obtained by CLX against *S. mutans*. Compound **1** also showed high in vitro TI values (6.82, 13.7, 3.41, 6.83 against *S. aureus*, *S. epidermidis*, *E. coli* and *L. species*, respectively). Additionally, the TI values of compound **10** against *S. aureus*, *S. epidermidis* and *S. mutans* were greater than those of compound **1**. Nevertheless, among all the tested agents, CLX exhibited the highest in vitro TI values. It should be noted that antibacterial agents which possess a value of therapeutic index higher than 10 can be administered to perform in vivo evaluation (Kashyap *et al.*, 2012; Ghareb *et al.*, 2012). Our two newly synthesized compounds (**1** and **6**) had antibacterial activity and exhibited excellent TI values higher than 10 against some bacterial strains.

### Antiproliferative activity evaluation

**Fig. 4:**
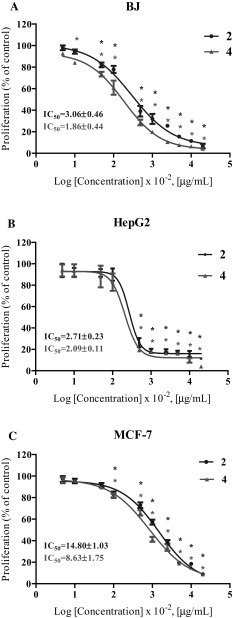
Antiproliferative activity of the synthesized compounds against normal human skin fibroblasts (**a**), human hepatocellular carcinoma (**b**) and human breast adenocarcinoma (**c**). The results were expressed as mean values ± SEM from three independent experiments. The IC_50_ values were presented as mean values ± SD.* Statistical significance obtained at *p* < *0.01* compared to the control

The synthesized compounds were also evaluated for in vitro antiproliferative activity against various cell lines, i.e., BJ (normal human skin fibroblasts), HepG2 (human hepatocellular carcinoma) and MCF-7 (human breast adenocarcinoma). In order to evaluate cell proliferation, the cells were treated with compounds at concentrations of 0.05, 0.1, 0.5, 1, 5, 10, 25, 50, 100 and 200 µg/mL for 96 h. Among the investigated compounds, only two (**2** and **4**) exhibited antiproliferative activity. Both compounds strongly decreased the BJ, HepG2 and MCF-7 cell proliferation in a concentration-dependent manner (Fig. 4).

The results were expressed as mean values ±SEM from three independent experiments. The IC_50_ values were presented as mean values ±SD. Statistical significance was obtained at *p* < 0.01 compared to the control. In the case of normal human cell line (BJ), compound **4** decreased cell proliferation more potently than compound **2**. The statistical significance for compound **4** against BJ cells was even obtained at 0.1 μg/mL (Fig. 4a).

Nevertheless, it should be noted that compound **4** suppressed the HepG2 and MCF-7 cell proliferation more effectively than compound **2**, with IC_50_ of 2.09 µg/mL (6.12 µM) and 8.63 µg/mL (25.3 µM), respectively (Fig. 4b, c).

Therefore, the IC_50_ value of compound **4** against the MCF-7 cell line was approximately two times lower than the IC_50_ value of compound **2**. On the other hand, compound **2** suppressed cell division of hepatocellular carcinoma slightly more potently than that of normal skin fibroblasts. The most pronounced effect was observed with 10 μg/mL of compound **2**, which reduced cell proliferation to 32 % (BJ) and to 18.9 % (HepG2) compared to the control (Fig. 4a, b). Thus, these data showed that compound **2** at 10 µg/mL is more effective against tumor than normal cells. According to the available literature data, our compounds exhibited a very high antiproliferative potential. The diarylthiosemicarbazide derivatives containing urea group and pyridine group at the para position, which also occur in the structure of our compounds, exhibited various antiproliferative activities against alveolar epithelial, lung and colorectal cell lines. After a 72-h incubation, the IC_50_ values of these compounds ranged from 1.8 to 82.4 μmol (Xin *et al.*, 2013). Moreover, Ghareb and colleagues reported that the thiosemicarbazide and semicarbazide derivatives of benzimidazole hydrazides with hydrazine hydrate afforded N^3^-substituted-5-((2-phenyl-1H-benzo[d]imidazol-1-yl)methyl)-4H-1,2,4-triazole-3,4-diamines that have antiproliferative potential against the MCF-7 cell line. Two of them after a 48-h exposure inhibited cell proliferation with an IC_50_ of 13.7 and 16.2 μg/mL (Ghareb *et al.*, 2012). Hence, our results indicated that the synthesized compounds **2** and **4** have a good antiproliferative potential against some tumor cells and may be promising candidates for further anticancer study.

### Structure–activity analysis

An important feature of a potential drug is its bioavailability which determines how an investigated compound can penetrate a biological membrane. Thus, the physiochemical analysis of a molecule known as Lipinski’s rule of five is used (Lipinski *et al.*, 1997). For this purpose, all thiosemicarbazides were analyzed in silico estimating their bioavailability via calculating such parameters as molecular weight (MW), partition coefficient (logP), the number of donors and acceptors of hydrogen bonds and the polar surface area (PSA). The obtained data showed that all compounds meet the criteria of Lipinski’s rule (Lipinski *et al.*, 1997). The molecular weight of the tested derivatives ranged from 290 to 385 Da (<500 Da), and the log *p* values ranged from −1.43 to 2.52 (<5), respectively. All the researched compounds have no more than five hydrogen bond donors (−NH and −OH) and fewer than ten hydrogen bond acceptors (N, O). This is very important information because a decreased number of donors are known to reduce the affinity of P-glycoprotein, and the more the acceptors, the more water molecules are connected. In addition, the amount of donors and acceptors of hydrogen bonds affect the magnitude of the compound’s polar surface area (PSA), which is defined as the sum of surfaces of polar atoms (usually of oxygen, nitrogen and attached hydrogen atoms) in a molecule. This is a useful parameter for the prediction of molecular transport properties, particularly in intestinal absorption and blood–brain barrier penetration (Fernandes and Gattass, 2009). Referring to our findings (Table 4), the PSA values of the tested thiosemicarbazide derivatives ranged from 92[Å^2^] to 118[Å^2^]. Compounds **2** and **4** have demonstrated the highest antibacterial and antiproliferative activities, and their PSA values were 92.5[Å^2^] and 94.1[Å^2^], respectively.

**Table 4:** Molecular properties of the thiosemicarbazides (**1–10**)

No	Ar	R	M.W. [amu]	logP	miLogP	n	n	PSA	TPSA
						ON	OHNH	[Å^2^]	
**1**	Pyridin-2-yl	2-FC_6_H_4_	290.32	1.46	1.087	5	3	93.5	66.044
**2**	**Pyridin-2-yl**	**2-ClC**_**6**_ **H**_**4**_	**306.77**	**1.89**	**1.601**	**5**	**3**	**92.5**	**66.044**
**3**	Pyridin-2-yl	CH_2_CH_2_morph.	385.48	−1.43	−0.213	7	3	111.0	78.516
**4**	**Pyridin-2-yl**	**2,4-Cl**_**2**_ **C**_**6**_ **H**_**3**_	**341.22**	**2.52**	**2.255**	**5**	**3**	**94.1**	**66.044**
**5**	Pyridin-2-yl	4-CH_3_SC_6_H_4_	317.41	2.04	1.405	5	3	117.7	66.044
**6**	Pyridin-3-yl	4-CH_3_SC_6_H_4_	317.41	1.56	1.339	5	3	117.0	66.044
**7**	Pyridin-3-yl	2,4-Cl_2_C_6_H_3_	341.22	2.05	2.189	5	3	93.8	66.044
**8**	Pyridin-4-yl	4-CH_3_SC_6_H_4_	317.41	1.56	1.289	5	3	117.1	66.044
**9**	Pyridin-4-yl	2-FC_6_H_4_	290.32	0.98	0.968	5	3	93.1	66.044
**10**	Pyridin-4-yl	2,4-Cl_2_C_6_H_3_	341.22	2.04	2.137	5	3	93.5	66.044

The obtained values of topological polar surface area (TPSA) confirmed this relationship (Table 4). It seems that this may be an important parameter for searching for a relation between structure and activity for this group of compounds.

## Conclusions

In this study, we reported the synthesis and antibacterial activity of new compounds with pyridinecarbonyl group connected to the thiosemicarbazide system. It should be noted that two thiosemicarbazide derivatives, i.e., **2** and **4,** exhibited good or moderate inhibition of all the most common caries-associated Gram-positive and Gram-negative bacterial strains. Moreover, these compounds strongly suppressed human hepatocellular carcinoma and human breast adenocarcinoma cell proliferation. The structure–activity relationship of the compounds showed that substitution at the position 2 of the pyridine ring enhances biological activity. The prominent antibacterial and antiproliferative effect of compounds **2** and **4** may be due to changing the number of chlorine atoms in the phenyl ring. Thus, it is worth underlying that 4-(2-chloro/2,4-dichlorophenyl)-1-(pyridine-2yl)carbonylthiosemicarbazide derivatives will be auspicious as potential agents for caries treatment and caries-associated cancer diseases. The physicochemical analysis indicates that the polar surface area is an important parameter for biological activity of the investigated compounds. Our results will have an impact on further investigation in this field in search of thiosemicarbazide compounds as antibacterial and antiproliferative agents.

## Experimental

### Chemicals and instruments

The chemicals used for synthesis and analysis were purchased from Merck Co. or Alfa Aesar and used without further purification. Melting points were determined on a Fisher-Johns block and presented without any corrections. The ^1^H and ^13^C NMR spectra were recorded on a Bruker Avance 300 MHz spectrometer in solution noted and with TMS as an internal standard. The IR spectra were recorded on a Thermo Nicolet 6700 ATR device in the range of 500–3500 cm^−1^. The elementary analysis was performed with the application of Perkin-Elmer analyzer (940 Winter St., Waltham, MA, USA). The obtained results were within ±0.4 % of the theoretical value. Follow-up of the reactions and the purity of the newly obtained compounds were checked using TLC on aluminum oxide 60 F_254_ plates (Merck) in a CHCl_3_/C_2_H_5_OH (10:1 and 10:2) solvent system with UV visualization. The carboxylic acid hydrazides were synthesized via the reaction of the appropriate carboxylic acid ester with 98 % hydrazine hydrate in the solution of anhydrous ethanol using the method described earlier (Idhayadhulla *et al.,*^2013^; Priebe *et al.*, 2008; Zamani, *et al.*, 2002).

### General procedure for the synthesis of 1-pyridinecarbonyl-4-substituted thiosemicarbazide derivatives (1–10)

A mixture of 2-, 3- or 4-pyridinecarboxylic acid hydrazide (0.01 mol), isothiocyanate (0.01 mol) and methanol (15 mL) was heated in a water bath reflux temperature for 0.5 h. The product was filtered, dried and crystallized from mixture methanol–acetonitrile (1:1).

#### *4*-*(2*-*Fluorophenyl)*-*1*-*(pyridin*-*2*-*yl)carbonylthiosemicarbazide* (**1**)

Yield 87 %, m.p. 182–184 °C. ^1^H NMR (DMSO-d_6_, 300 MHz) δ: 7.16–7.66 (m, 4H, CH_phenyl_), 8.01–8.69 (m, 4H, CH_pyridine_), 9.52 (s, 1H, –NH exchangeable with D_2_O), 9.88 (s, 1H, NH exchangeable with D_2_O), 10.81 (s, 1H, NH exchangeable with D_2_O). ^13^C NMR (DMSO-d_6_, 75 MHz) δ: 116.01, 116.14, 122.98, 124.30, 127.41, 128.34, 130.87, 138.12, 148.95, 149.92, 164.25, 182.48. FT-IR ν: 3312, 1658, 1351 cm^−1^. MS (Cl) *m/z* = 291 [M^+^]. Anal.: Calcd. for C_13_H_11_N_4_OSF (290.31): C (53.78), H (3.82), N (19.29). Found: C (53.81), H (3.87), N (19.19) (CAS: 891086-61-6).

#### *4*-*(2*-*Chlorophenyl)*-*1*-*(pyridin*-*2*-*yl)carbonylthiosemicarbazide* (**2**)

Yield 83 %, m.p. 172–174 °C. ^1^H NMR (DMSO-d_6_, 300 MHz) δ: 7.25–7.66 (m, 4H, CH_phenyl_), 8.02–8.71 (m, 4H, CH_pyridine_), 9.54 (s, 1H, –NH exchangeable with D_2_O); 9.88 (s, 1H, NH exchangeable with D_2_O); 10.82 (s, 1H, NH exchangeable with D_2_O). ^13^C NMR (DMSO-d_6_, 75 MHz) δ: 122.99, 127.47, 128.18, 129.74, 131.00, 138.17, 149.01, 149.87, 164.27, 182.17. FT-IR ν: 3246, 1655, 1354 cm^−1^. MS (CI) *m/z*: 307 (M^+^). Anal.: Calcd. for C_13_H_11_N_4_OSCl (306.77): C (50.89), H (3.61), N (18.29). Found: C (50.91), H (3.64), N (18.33) (CAS: 894234-77-6).

#### *4*-*(2*-*Morpholinoethyl)*-*1*-*(pyridin*-*2*-*yl)carbonylthiosemicarbazide* (**3**)

Yield 90 %, m.p. 196–198 °C. ^1^H NMR (DMSO-d_6_, 300 MHz) δ: 2.35–2.59 (m, 4H, 2xCH_2_ morpholine), 3.45–3.52 (4H, 2xCH_2_ morpholine), 3.57–3.58 (m, 2H, –NH–CH_2_–CH_2_–), 3.74–3.76 (m, 2H, –NH–CH_2_–CH_2_–), 7.64–8.69 (m, 4H, CH_pyridine_), 8.69 (s, 1H, NH exchangeable with D_2_O), 9.43 (s, 1H, NH exchangeable with D_2_O), 10.63 (s, 1H, NH exchangeable with D_2_O). ^13^C NMR (DMSO-d_6_, 75 MHz) δ: 53.22, 53.59, 53.82, 56.50, 56.89, 57.27, 66.44, 66.55, 66.68, 122.94, 127.50, 138.23, 149.03, 149.66, 181.70. FT-IR ν: 3292, 1627, 1338 cm^−1^. MS (CI) *m/z*: 308 (M+). Anal.: Calcd. for C_13_H_19_N_5_O_2_S (309.38): C (50.46), H (6.18), N (22.63). Found: C (50.51), H (6.22), N (22.57) (CAS: 455314-30-4).

#### *4*-*(2,4*-*Dichlorophenyl)*-*1*-*(pyridin*-*2*-*yl)carbonylthiosemicarbazide* (**4**)

Yield 91 %, m.p. 158–160 °C. ^1^H (DMSO-d_6_, 300 MHz) δ: 7.42–7.66 (m, 3H, CH_phenyl_), 8.02–8.69 (m, 4H, CH_pyridine_), 9.57 (s, 1H, NH exchangeable with D_2_O), 9.96 (s, 1H, NH exchangeable with D_2_O), 10.83 (s, 1H, NH exchangeable with D_2_O). ^13^C NMR (DMSO-d_6_, 75 MHz) δ: 123.06, 127.46, 127.68, 129.20, 131.72, 132.52, 136.70, 138.15, 148.97, 164.27, 182.35. FT-IR ν: 3242, 3108, 1652, 1346 cm^−1^. MS (CI) *m/z*: 342 (M^+^). Anal.: Calcd. for C_13_H_10_N_4_OSCl_2_ (341.21): C (45.75), H (2.95), N (16.41). Found: C (45.80), H (2.97), N (16.37) (CAS: 891538-65-4).

#### *4*-*(4*-*Methylthiophenyl)*-*1*-*(pyridin*-*2*-*yl)carbonylthiosemicarbazide* (**5**)

Yield 88 %, m.p. 184–186 °C. ^1^H NMR (DMSO-d_6_, 300 MHz) δ: 2.28 (s, 3H, CH_3_), 7.01–7.25 (m, 4H, CH_phenyl_), 7.51–8.25 (m, 4H, CH_pyridine_), 8.66 (1 s, 1H, NH exchangeable with D_2_O), 10.53 (s, 1H, NH exchangeable with D_2_O), 12.01 (s, 1H, NH exchangeable with D_2_O). ^13^C NMR (DMSO-d_6_, 75 MHz) δ: 14.79, 124.50, 125.47, 125.89, 129.26, 132.24, 137.81, 139.69, 145.62, 149.68, 150.00, 169.69. FT-IR ν: 3236, 3111, 1654, 1324 cm^−1^. MS (CI) *m/z*: 318 (M^+^). Anal.: Calcd. for C_14_H_14_N_4_OS_2_ (318.41): C (52.80), H (4.43), N (17.59). Found: C (52.71), H (4.38), N (17.51).

#### *4*-*(4*-*Methylthiophenyl)*-*1*-*(pyridin*-*3*-*yl)carbonylthiosemicarbazide* (**6**)

Yield 89 %, m.p. 176–177 °C. ^1^H NMR (DMSO-d_6_, 300 MHz) δ: 2.47 (s, 3H, CH_3_), 7.23–7.57 (m, 4H, CH_phenyl_), 8.27–8.76 (m, 4H, CH_pyridine_), 9.11 (s, 1H, NH exchangeable with D_2_O), 9.81 (s, 1H, NH exchangeable with D_2_O), 10.76 (s, 1H, NH exchangeable with D_2_O). ^13^C NMR (DMSO-d_6_, 75 MHz) δ: 15.58, 123.90, 126.24, 127.14, 128.72, 134.96, 136.05, 136.83, 149.40, 152.84, 165.14, 181.53. FT-IR ν: 3284, 1632, 1341 cm^−1^. MS (CI) *m/z* (%): 319 (M^+^). Anal.: Calcd. for C_14_H_14_N_4_OS_2_ (318.41): C (52.80), H (4.43), N (17.59). Found: C (52.72), H (4.49), N (17.64).

#### *4*-*(2,4*-*Dichlorophenyl)*-*1*-*(pyridin*-*3*-*yl)carbonylthiosemicarbazide* (**7**)

Yield 92 %, m.p. 196–197 °C. ^1^H (DMSO-d_6_, 300 MHz) δ: 7.37–7.68 (m, 3H, CH_phenyl_), 8.27–9.11 (m, 4H, CH_pyridine_), 9.75 (s, 1H, NH exchangeable with D_2_O), 10.02 (s, 1H, NH exchangeable with D_2_O), 10.86 (s, 1H, NH exchangeable with D_2_O). ^13^C NMR (DMSO-d_6_, 75 MHz) δ: 123.96, 129.36, 135.14, 148.55, 152.23, 164.80. FT-IR ν: 3331, 3150, 1700, 1359 cm^−1^. MS (CI) *m/z*: 342 (M^+^). Anal.: Calcd. for C_13_H_10_N_4_OSCl_2_ (341.21): C (45.75), H (2.95), N (16.41). Found: C (45.98), H (2.91), N (16.52) (CAS: 475180-05-3).

#### *4*-*(4*-*Methylthiophenyl)*-*1*-*(pyridin*-*4*-*yl)carbonylthiosemicarbazide* (**8**)

Yield 86 %, m.p. 197–198 °C. ^1^H NMR (DMSO-d_6_, 300 MHz) δ: 2.47 (s, 3H, CH_3_), 7.23–7.86 (m, 4H, CH_phenyl_), 8.77–8.78 (m, 4H, CH_pyridine_), 9.83 (s, 2H, NH exchangeable with D_2_O), 10.86 (s, 1H, NH exchangeable with D_2_O). ^13^C NMR (DMSO-d_6_, 75 MHz) δ: 15.57, 122.15, 126.26, 127.04, 134.97, 136.79, 140.07, 150.67, 164.93, 181.45. FT-IR ν: 3097, 2936, 1667, 1378 cm^−1^. MS (CI) m/z (%): 319 (M^+^). Anal.: Calcd. for C_14_H_14_N_4_OS_2_ (318.41): C (52.80), H (4.43), N (17.59). Found: C (52.96), H (4.51), N (52.68).

#### *4*-*(2*-*Fluorophenyl)*-*1*-*(pyridin*-*4*-*yl)carbonylthiosemicarbazide* (**9**)

Yield 78 %, m.p. 202–204 °C. ^1^H NMR (DMSO-d_6_, 300 MHz) δ: 7.18–7.31 (m, 4H, CH_phenyl_), 7.86–8.78 (m, 4H, CH_pyridine_), 9.70 (s, 1H, NH exchangeable with D_2_O), 9.99 (s, 1H, exchangeable with D_2_O), 10.94 (s, 1H, NH exchangeable with D_2_O). ^13^C NMR (DMSO-d_6_, 75 MHz) δ: 116.14, 116.27, 122.21, 124.44, 127.51, 128.74, 131.17, 140.00, 150.65, 157.08, 158.70, 165.02, 182.69. FT-IR ν: 3265, 3113, 1677, 1368 cm^−1^. MS (CI) *m/z*: 291 (M^+^). Anal.: Calcd. for C_13_H_11_N_4_OSF (290.31): C (53.78), H (3.82), N (19.29). Found: C (53.65), H (3.74), N (19.42) (Byung *et al.*, 2004).

#### *4*-*(2,4*-*Dichlorophenyl)*-*1*-*(pyridin*-*4*-*yl)carbonylthiosemicarbazide* (**10**)

Yield 84 %, m.p. 164–166 °C. ^1^H NMR (DMSO-d_6_, 300 MHz) δ: 7.37–7.45 (m, 3H, CH_phenyl_), 7.68–8.78 (m, 4H, CH_pyridine_), 9.76 (s, 1H, NH exchangeable with D_2_O), 10.05 (s, 1H, NH exchangeable with D_2_O), 10.95 (s, 1H, NH exchangeable with D_2_O). ^13^C NMR (DMSO-d_6_, 75 MHz) δ: 122.24, 127.81, 129.30, 132.12, 132.92, 133.27, 136.55, 150.65, 165.09, 182.53. FT-IR ν: 3309, 3117, 1677, 1380 cm^−1^. MS (CI) *m/z* (%): 341 (M^+^). Anal.: Calcd. for C_13_H_10_N_4_OSCl_2_ (341.21): C (45.75), H (2.95), N (16.41). Found: C (45.69), H (2.90), N (17.01) (Goldfarb, 2009).

### X-ray analysis

The X-ray diffraction intensities were collected at 100 K on an Oxford Diffraction Xcalibur CCD diffractometer with graphite-monochromatized MoKα radiation (λ = 0.71073 Å) using the ω scan technique, with an angular scan width of 1.0°. The programs CrysAlis CCD and CrysAlis Red (Oxford Diffraction, Xcalibur CCD System, CRYSALIS Software System, Version 1.171, Oxford Diffraction Ltd. 2009) were used for data collection, cell refinement and data reduction. Absorption corrections were applied using the multi-scan method by Blessing (Blessing, 1995). The structures were solved via direct methods using SHELXS-97 and refined by the full-matrix least-squares on *F*^2^ using the SHELXL-97 (Sheldrick, 2008). Non-hydrogen atoms were refined with anisotropic displacement parameters. The N-bonded H atoms were found in the difference Fourier maps and then remained fixed during the least-squares refinements. All the remaining H atoms were positioned geometrically and allowed to ride on their parent atoms, with U_iso_(H) = 1.2 U_eq_(C). The molecular plots were drawn with Olex2 (Dolomanov *et al.*, 2009).

### Antibacterial activity

Panel reference strains of bacteria from the American Type Culture Collection or Polish Collection of Microorganisms, including aerobic Gram-positive bacteria: *Staphylococcus aureus* ATCC 25923 and *Staphylococcus epidermidis* ATCC 12228, and aerobic Gram-negative bacteria: *Escherichia coli* ATCC 25922 and *Pseudomonas aeruginosa* ATCC 9027, as well as microaerobic Gram-positive bacteria: *Lactobacillus* spp., *Lactobacillus acidophilus* PCM 2105, *Streptococcus mutans* PCM 2502 and *Streptococcus sanguinis* PCM 2335, were used. Microbial suspensions with an optical density of 0.5 McFarland standard at 1.5 × 108 CFU/mL (CFU: colony forming unit) were prepared in sterile 0.9 % NaCl. Mueller–Hinton (M–H) broth and M–H agar (Oxoid Ltd., England) for aerobic strains, and MRS Broth Lactobacillus, MRS Agar Lactobacillus (BioMaxima S.A., Poland), BHI Broth and BHI agar (BioMaxima S.A., Poland) for microaerobic strains were used in the microbial tests. All stock solutions of the newly synthesized compounds were prepared in DMSO (the final DMSO concentration used in bacterial tests did not inhibit microbial growth and was less than 1.5 %). The antibacterial activity of the newly synthesized compounds was compared with the controls: cefepime dihydrochloride (Maxipime, Bristol-Myers Squibb Latina), chlorhexidine digluconate ((CLX) Amara Poland) and ethacridine lactate (Rivanolum, PharmaSwiss, Czech Republic).

### Disk diffusion method

The preliminary antibacterial activity of the carbazide derivatives against human pathogenic Gram-positive, Gram-negative aerobic and microaerobic bacteria was evaluated by measuring the zones of inhibition in the disk diffusion method (Murray *et al.*, 1995). Each compound (100 µg) was placed on Petri plates with agar medium (previously inoculated with 0.5 McFarland standards with the tested bacterial strains). After 18 h of incubation at 37 °C (for aerobic strains) or 40 h at 35 °C (for microaerobic strains), zones of microbial growth produced around the tested substances were measured and recorded as the diameters of inhibition.

### Broth microdilution method

A broth microdilution method was used to evaluate the minimum inhibition concentration (MIC) according to the CLSI document (CLSI performance standards for antimicrobial susceptibility testing, 2008, Eighteenth International Supplement, CLSI document M7-MIC, Clinical Laboratory Standards Institute, Wayne) with some modifications. The lowest concentration of the tested compound (expressed in μg/mL) which did not allow any visible growth of bacteria was considered as MIC. A serial doubling dilution of the compounds was prepared in 96-well plates (200 μL per well). A suitable medium (M-H Broth, MRS Broth Lactobacillus, BHI Broth) was used as a diluent. The final concentrations of derivatives were 1000–0.015 μg/mL. Finally, 2 μL of inoculum of the tested bacterial strain (1.5 × 108 CFU/mL) was added to each well. The tests were performed either at 36 °C for 18 h (aerobic strains) or at 40 h (microaerobic strains). After incubation, the panel was digitally analyzed at 600 nm using the microplate reader Bio Tech Synergy (USA) with a dedicated software system. The growth intensity in each well was compared with the negative and positive controls.

### Cell lines

Normal human skin fibroblasts (BJ), human hepatocellular carcinoma (HepG2) and human breast adenocarcinoma (MCF-7) were obtained from American Type Culture Collection (ATCC, England, UK). The cells were cultured in Eagle’s minimum essential medium (EMEM, ATCC) supplemented with 10 % fetal bovine serum (FBS, PAA Laboratories), 100 U/mL penicillin and 100 μg/mL streptomycin (Sigma-Aldrich). In the case of the MCF-7 cell line, the culture medium was additionally supplemented with 0.01 μg/mL of human recombinant insulin (Sigma-Aldrich). The cells were grown in 75-cm^2^ flasks and maintained at 37 °C in a humidified atmosphere of 5 % CO_2_ and 95 % air.

### Cytotoxicity assay

In order to determine the cytotoxicity, BJ cells were seeded in flat-bottom 96-well plates in 100 μL of a complete growth medium at a concentration of 1.7 × 104 cells/well and incubated for 24 h at 37 °C in a humidified atmosphere of 5 % CO_2_. Immediately before drug treatment, the synthesized compounds (**1**, **2**, **4**, **5**, **6**, **7**, **10**) were dissolved in dimethyl sulfoxide (DMSO, Sigma-Aldrich) and then diluted in cell culture medium supplemented with 2 % FBS. Moreover, cefepime dihydrochloride (Maxipime, Bristol-Myers Squibb Latina), chlorhexidine digluconate ((CLX) Amara Poland) and ethacridine lactate (Rivanolum, PharmaSwiss, Czech Republic) were used as reference antibacterial agents. After incubation, the growth medium was replaced with 100 μL of the appropriate serial dilutions of the investigated compounds. Untreated cells were used as negative controls, and different concentrations of DMSO were used as the solvent control. The cell cultures were incubated at 37 °C for 24 h. The cytotoxicity was estimated using the 3-(4,5-dimethylthiazol-2-yl)-2,5-diphenyltetrazolium bromide (MTT) assay as described by Mosmann with some modifications (Mosmann, 1983). Briefly, the cells were incubated for 3 h with 25 μL of MTT solution (5 μg/mL in PBS buffer) per well. The MTT assay is a rapid colorimetric method based on the conversion of mitochondrial succinate dehydrogenase from yellow, soluble tetrazolium salt to blue formazan crystals to determine the number of viable cells. In order to dissolve formazan crystals in live cells, 100 μL of SDS-HCl solution (10 % SDS in 0.01 N HCl) was added per well. After overnight incubation, absorbance was measured at 570 nm using microplate reader (BioTek ELx50). The MTT assay was repeated in three independent experiments performed in octuplicates. The half-maximal cytotoxic concentration (CC_50_) was defined as the compound concentration (μg/mL) required to reduce cell viability to 50 %. Therapeutic index (TI) is a widely accepted parameter to represent the specificity of antibacterial agents for human (Begg *et al.*, 1999). In this study, the in vitro TI values were calculated as the ratio of CC_50_ (cytotoxic activity) and MIC (antibacterial activity); thus, greater values of in vitro therapeutic index indicate safer specificity for eukaryotic cells.

### Cell proliferation assay

In order to evaluate cell proliferation, the cells were seeded in flat-bottom 96-well plates in 100 μL of a complete growth medium at a concentration of 2 × 103 cells/well (BJ), 1.5 × 104 cells/well (HepG2) and 2.5 × 104 cells/well (MCF-7) and incubated for 24 h at 37 °C in a humidified atmosphere of 5 % CO_2_. Before drug exposure, the synthesized compounds (**1–10**) were dissolved in DMSO and then diluted in a complete culture medium supplemented with 10 % FBS. Subsequently, the growth medium was gently removed and the cells were exposed to 100 μL of serial dilutions of the investigated compounds at concentrations ranging from 0.05 to 200 μg/mL. Untreated cells were used as negative controls, and different concentrations of DMSO were used as the solvent control. After 96-h incubation at 37 °C in a humidified atmosphere of 5 % CO_2_, cell proliferation was assessed using the MTT test as described previously (Cytotoxicity assay). The MTT assay was repeated in three independent experiments in quadruplicates. The half-maximal inhibitory concentration (IC_50_) was defined as the compound concentration (μg/mL) required to inhibit cell proliferation to 50 %.

### Statistical analysis

The results of the in vitro cell culture experiments were presented as mean values ± standard deviation (SD) or as mean values ± standard error of the mean (SEM). The data were analyzed using one-way ANOVA test followed by Dunnett’s test. Differences were considered as significant with *p* < 0.01 (GraphPad Prism 5, Version 5.04 Software). The values of CC_50_ and IC_50_ were calculated via 4-parameter nonlinear regression analyses using GraphPad Prism 5, version 5.04.

### Molecular modeling

Molecular modeling was performed using generally available software. The LogP and PSA parameters were performed by using VEGA ZZ program (Pedretti *et al.*, 2004). The geometry and energy of the tested compounds were optimized by AM1 semiempirical method (Dewar *et al.*, 1985). The TPSA, miLogP and hydrogen bond donors and acceptors were calculated by Molinspiration program (http://www.molinspiration.com/cgi-bin/properties-accessed 1 February, 2015).

## Electronic supplementary material

Below is the link to the electronic supplementary material.

Supplementary material 1 (DOC 253 kb)
