# Supplementary material for: Synthesis, antibacterial and antiproliferative potential of some new 1-pyridinecarbonyl-4-substituted thiosemicarbazide derivatives
Source: Med Chem Res. 2016 Jun 1;25:1666–77. doi: 10.1007/s00044-016-1599-6 (PMC4958397; doi:10.1007/s00044-016-1599-6)
Supplement: Supplementary file 1 — Supplementary material 1 (DOC 253 kb) [file 44_2016_1599_MOESM1_ESM.doc]

**Suplementary material**

**Synthesis, antibacterial and antiproliferative potential of some new**

**1-pyridinecarbonyl-4-substituted thiosemicarbazide derivatives**

Monika Pitucha, Maciej Wos, Malgorzata Miazga-Karska, Katarzyna Klimek, Barbara Miroslaw, Anna Pachuta-Stec, Agata Gładysz, Grazyna Ginalska

**Comp. 1 -1H NMR**

**
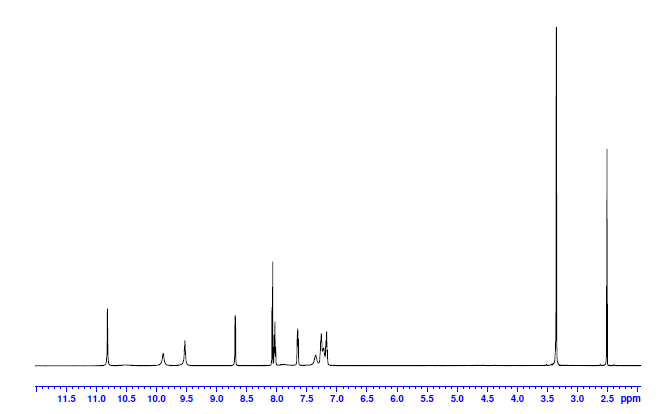
**

**Comp. 2 – 1H NMR
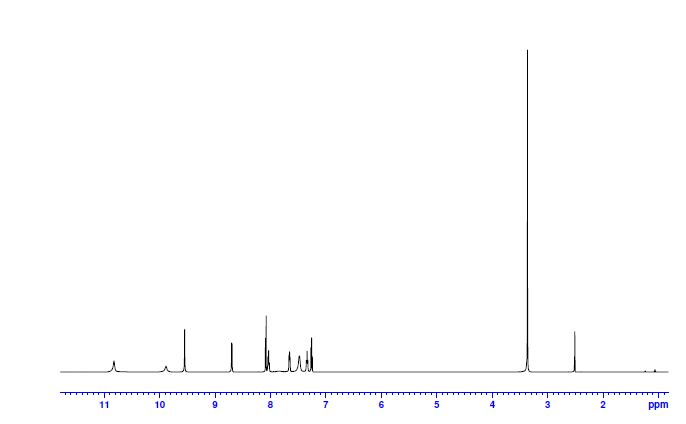
**

**Comp. 3 – 1H NMR**

**
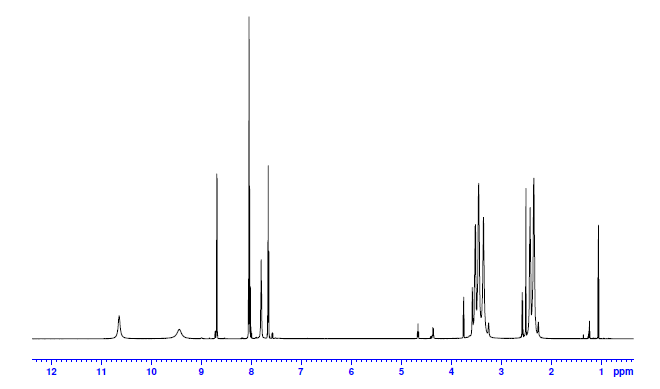
**

**Comp. 4 – 1H NMR**

**
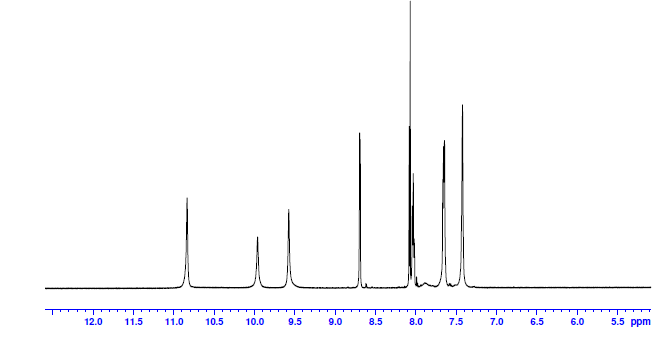
**

**Comp. 5 – 1H NMR**

**
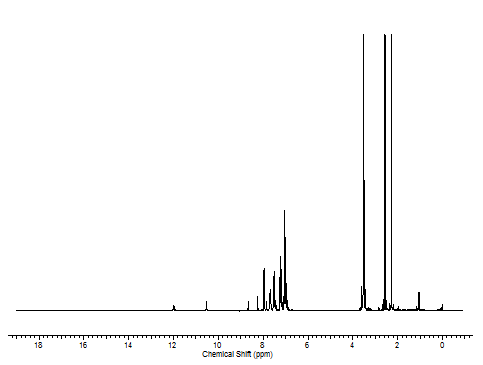
**

**Comp. 6 – 1H NMR**

**
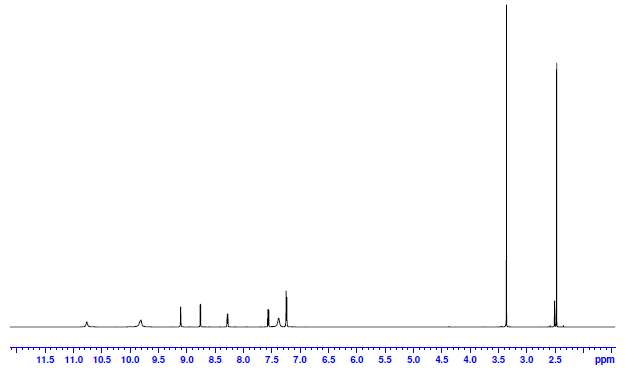
**

**Comp. 7 – 1H NMR**

**
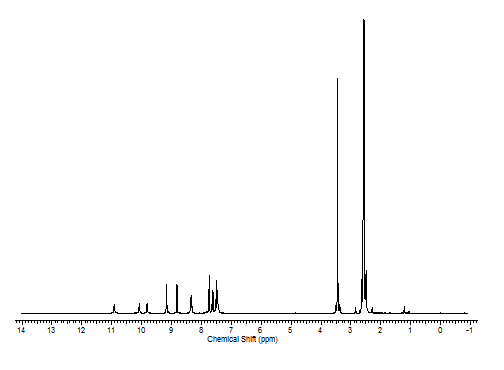
**

**Comp. 8 – 1H NMR**

**
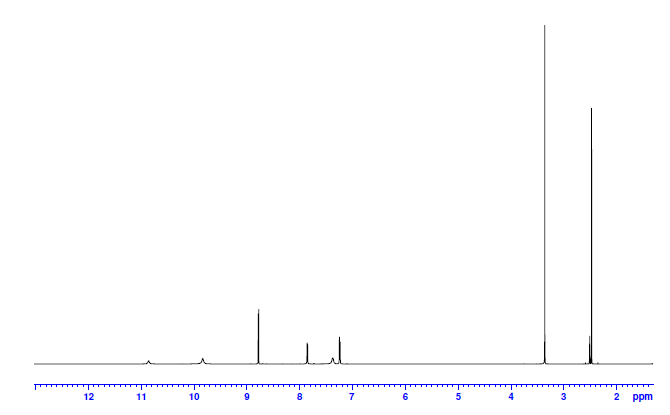
**

**Comp. 9 -1H NMR**

**
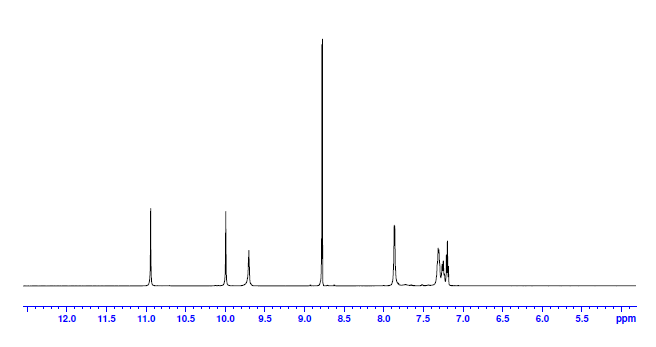
**

**Comp. 10 – 1H NMR**

**
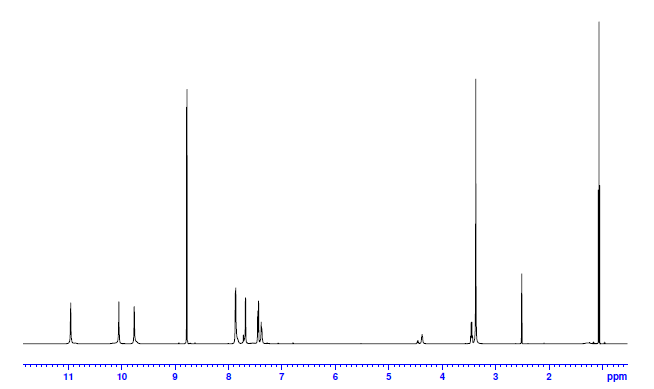
**

**Comp. 1 – 13C NMR**

**Comp. 2 – 13C NMR**

**Comp. 3 – 13C NMR**

**
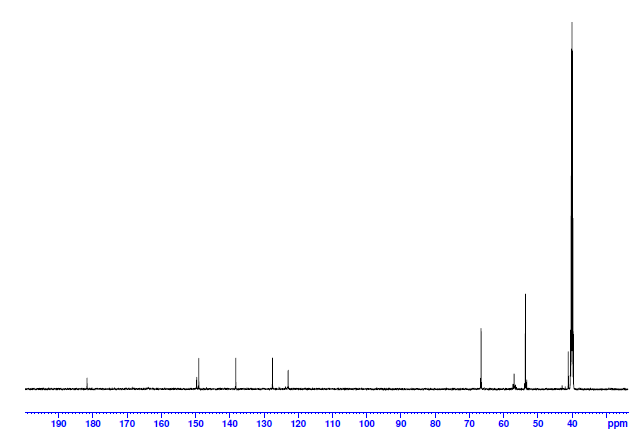
**

**Comp. 4 – 13C NMR**

**
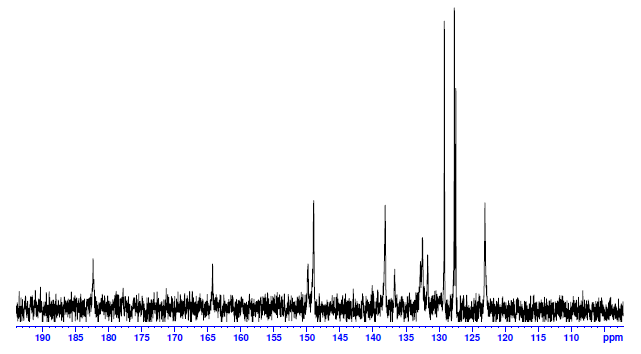
**

**Comp. 5 – 13 C NMR**

**
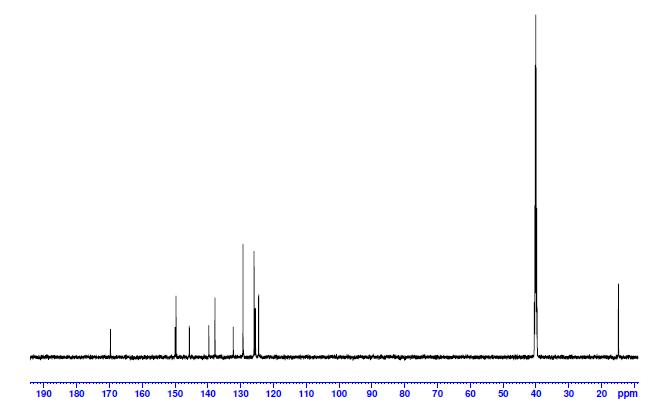
**

**Comp. 6 – 13C NMR**

**
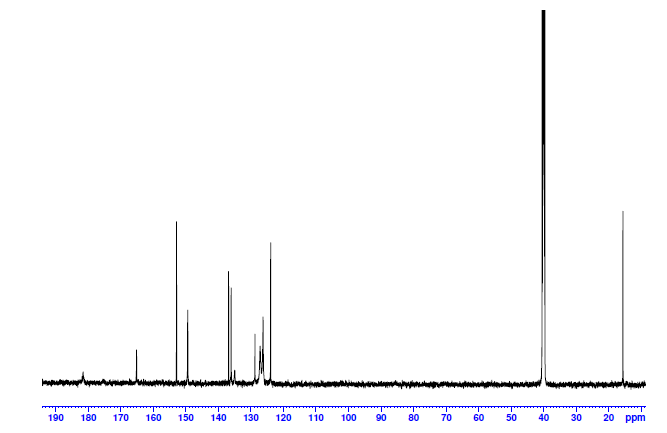
**

**Comp. 8 – 13C NMR**

**
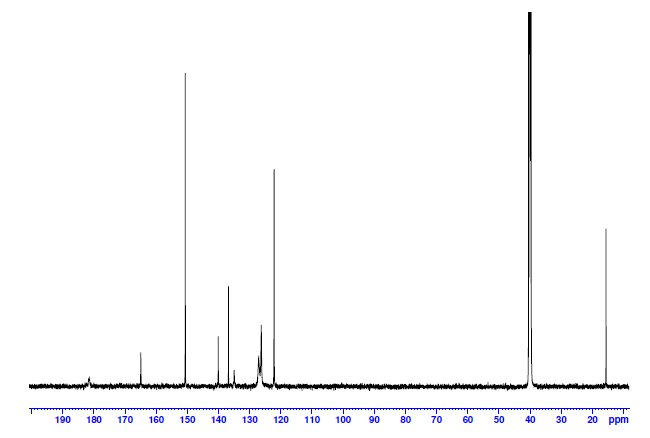
**

**Comp. 9 – 13C NMR**

**Comp. 10 -13 NMR**

**
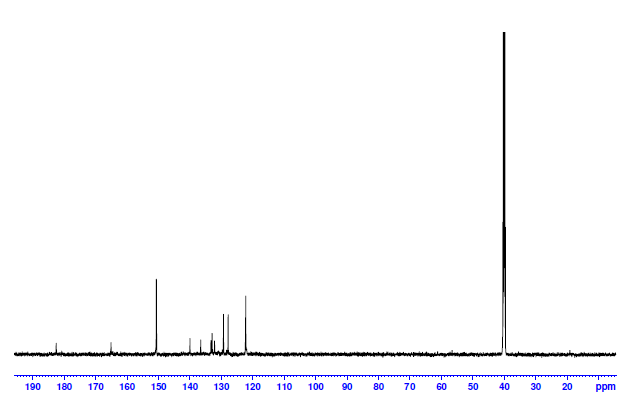
**
